# Transformer models in biomedicine

**DOI:** 10.1186/s12911-024-02600-5

**Published:** 2024-07-29

**Authors:** Sumit Madan, Manuel Lentzen, Johannes Brandt, Daniel Rueckert, Martin Hofmann-Apitius, Holger Fröhlich

**Affiliations:** 1 Department of Bioinformatics, Fraunhofer Institute for Algorithms and Scientific Computing (SCAI), Schloss Birlinghoven, Sankt Augustin, 53757 Germany; 2 Institute of Computer Science, University of Bonn, Bonn, 53115 Germany; 3 Bonn-Aachen International Center for Information Technology (B-IT), University of Bonn, Bonn, 53115 Germany; 4 School of Medicine, Klinikum Rechts der Isar, Technical University Munich, Munich, Germany; 5 School of Computation, Information and Technology, Technical University Munich, Munich, Germany; 6 Department of Computing, Imperial College London, London, UK

**Keywords:** Transformer, Biomedicine, Life Science, Deep learning, Neural networks, Machine learning

## Abstract

Deep neural networks (DNN) have fundamentally revolutionized the artificial intelligence (AI) field. The transformer model is a type of DNN that was originally used for the natural language processing tasks and has since gained more and more attention for processing various kinds of sequential data, including biological sequences and structured electronic health records. Along with this development, transformer-based models such as BioBERT, MedBERT, and MassGenie have been trained and deployed by researchers to answer various scientific questions originating in the biomedical domain. In this paper, we review the development and application of transformer models for analyzing various biomedical-related datasets such as biomedical textual data, protein sequences, medical structured-longitudinal data, and biomedical images as well as graphs. Also, we look at explainable AI strategies that help to comprehend the predictions of transformer-based models. Finally, we discuss the limitations and challenges of current models, and point out emerging novel research directions.

## Introduction

The transformer [1] is a well-known deep neural network (DNN) model, which has revolutionized the artificial intelligence (AI) field. The architecture of the transformer builds the backbone of large language models (LLM), enabling them to harness the power of vast amounts of data to gain a more profound understanding of the underlying information. The architecture was initially developed for comprehending natural language, achieving this by analyzing every input sentence and capturing the context of each word through focusing on other words. Generic LLMs have brought significant advancements to various natural language processing (NLP) tasks ranging from machine translation over text generation to question answering. Most common examples of generic LLMs include Generative Pre-trained Transformer (GPT) [2], Bidirectional Encoder Representations from Transformers (BERT) [3], Large Language Model Meta AI (LLamA) [4, 5], and BigScience Large Open-science Open-access Multilingual Language Model (BLOOM) [6].

**Fig. 1:**
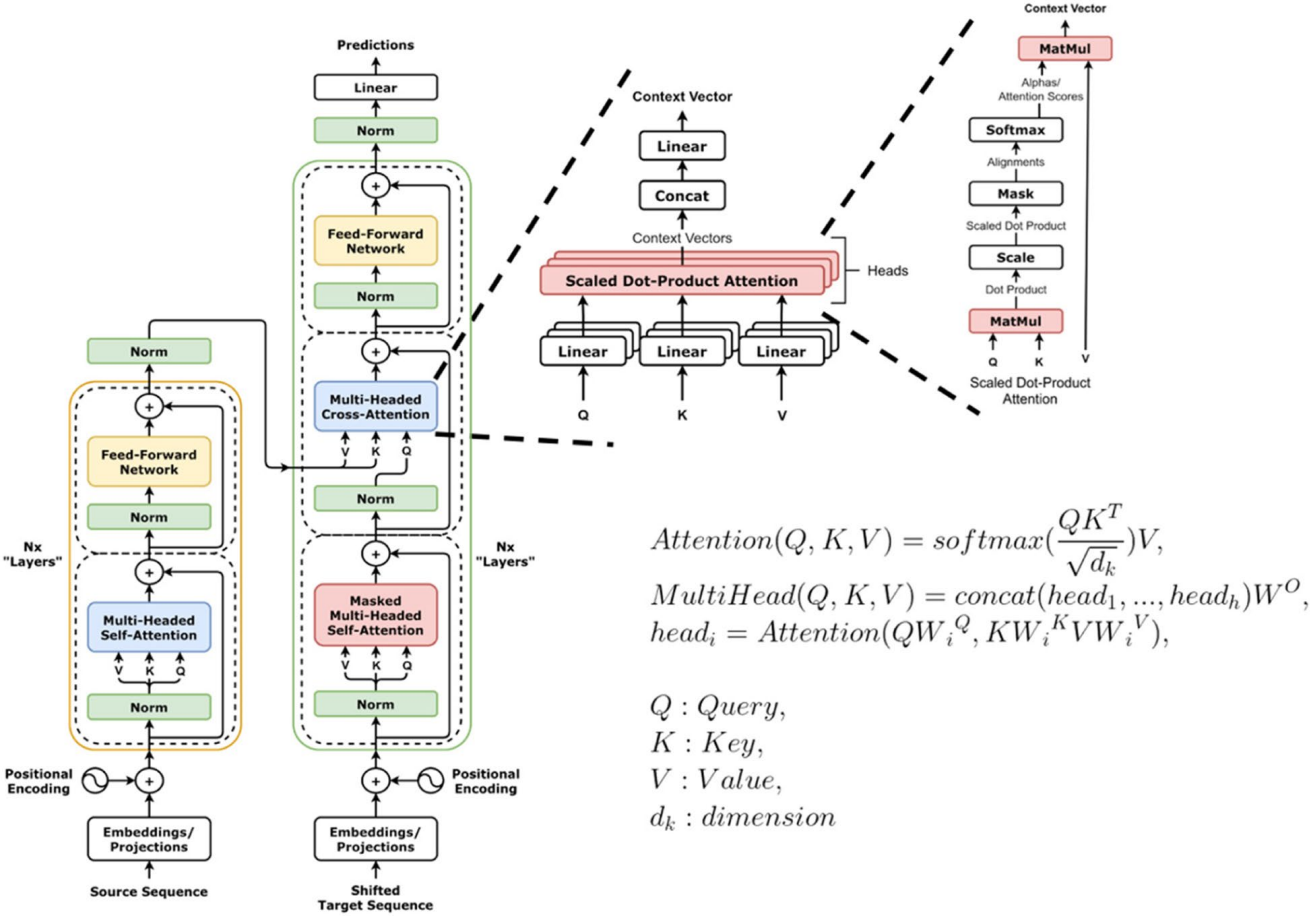
The transformer architecture with its self-attention mechanism. Original transformer images by https://github.com/dvgodoy/dl-visuals / CC BY 4.0.)

The success of transformer-based models can be attributed to the self-attention mechanism, integrated encoder-decoder architecture, and scalable as well as modular structure. These characteristics allow it to learn effective representations of the underlying data, encode long-range dependencies, and process huge amounts of data in an efficient way. The basic building block of the transformer is the self-attention mechanism [1, 7]. This mechanism allows the model to learn complex sequence representations by incorporating or attending to the information throughout the other parts of the same sequence. Equally important is the encoder-decoder structure of the transformer while both comprise multiple layers and variants of the self-attention mechanism (Fig. 1). This type of architecture facilitates sequence-to-sequence learning; therefore, transformers were originally used to solve the machine translation problem (e.g., translation from English to German). The encoder-only architecture (for instance, utilized in BERT) can be used for classification and understanding tasks [3], whereas decoders-only (such as GPT, LLamA, and BLOOM) are used for generative tasks [8, 9]. Furthermore, the modular and scalable architecture of the transformer allows the stacking of encoder and decoder blocks on each other, which substantially increases the capacity of the model. By processing huge amounts of data with larger models, the performance of transformers has been significantly increased on various tasks [8].

Transformer-based models such as BERT or GPT apply a two step process in their approach to understand the data provided to them and handling various downstream tasks. In a pre-training phase, they leverage the abundant unlabeled data to learn a general representation through an embedding model of the underlying objects in a self-supervised manner. Unlabeled data, characterized by the absence of labels or tags, is widely available. For instance, the web contains a vast amount of textual content in the form of web pages, blogs, and forums that are not categorized or labeled. In contrast, labeled datasets contain data that have been annotated with specific labels or categories, such as the label “gene” in case of biomedical texts. Due to the manual annotation process, obtaining labeled data is often more challenging and time-consuming compared to unlabeled data. In the fine-tuning phase, the pre-trained general representation model is used to train a supervised use case-specific task model using the limited labeled data. Over time they have been applied successfully beyond language to process other modalities and brought significant advancements to speech processing, computer vision (CV), and many more areas.

**Fig. 2:**
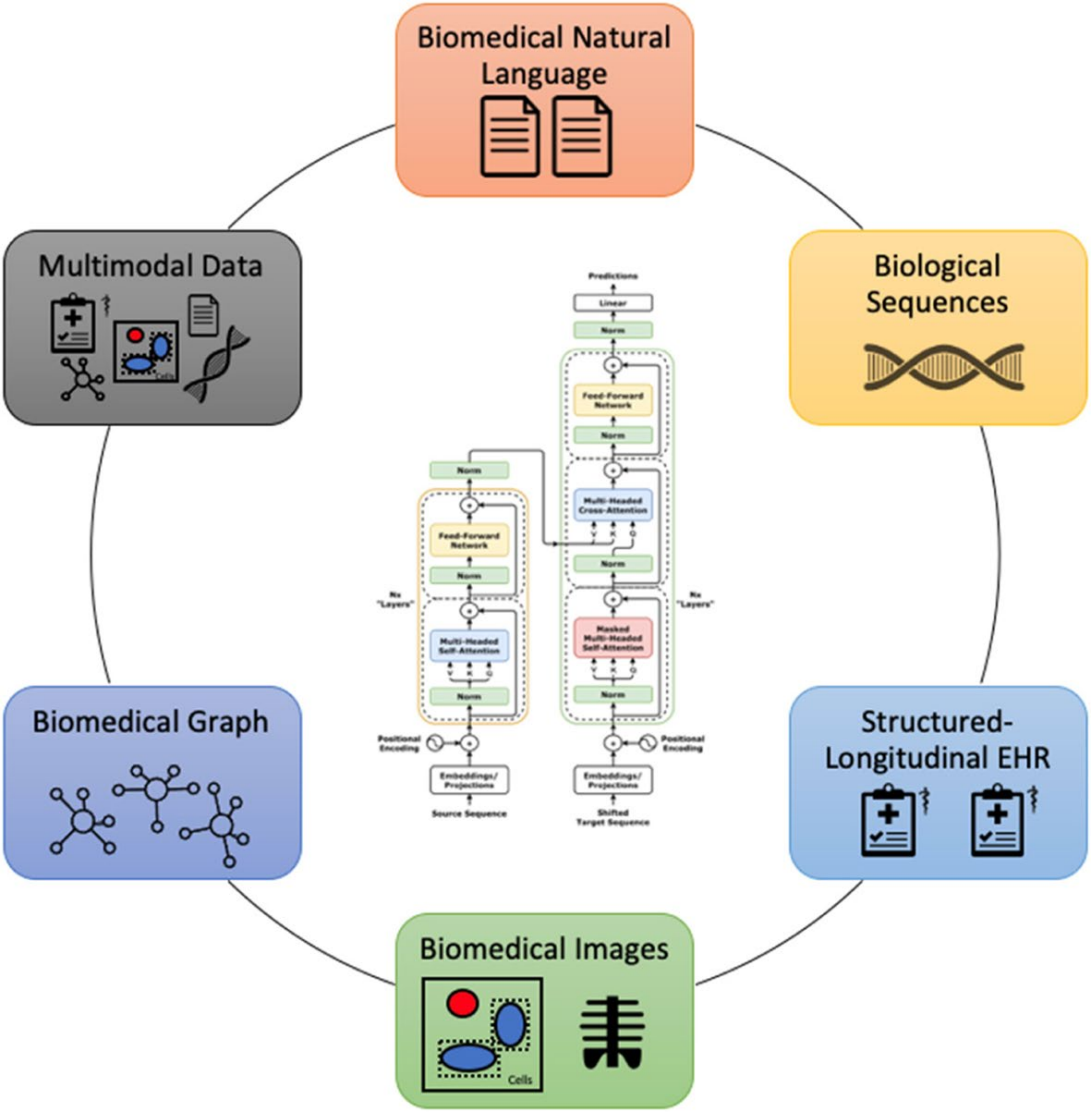
Application fields of transformers in biomedicine. Transformer image by https://github.com/dvgodoy/dl-visuals / CC BY 4.0

Transformers are now in the spotlight of many areas of biomedical-related AI research. They have been proven instrumental in addressing diverse biomedical-related questions, facilitating the analysis of data modalities ranging from biomedical literature to complex imaging and genetic information. The pace of progress has reached a limit that is difficult to grasp and, therefore, requires a thorough survey of the field. To our knowledge, such a thorough review is missing so far. Our paper thus tries to fill a gap. In the following, we highlight and discuss transformer-based models in five application fields (Fig. 2): 1) biomedical natural language processing (including biomedical literature, clinical notes, and social media text), 2) biological sequences (including protein sequences), 3) structured-longitudinal electronic health records (EHR), 4) biomedical images, and 5) biomedical graphs. We also introduce some studies that have pursued learning on multiple modalities jointly. Finally, we discuss methods to make transformer-based predictions, and we conclude by providing a prospect for future research.

Table 1 provides a glossary of concepts of AI that are discussed in this work. The mathematical details on transformers will not be elaborated in this work, however, we refer the readers to [10, 11] for more details.

**Table 1:** Glossary of AI and machine learning terminology sorted alphabetically

Concept	Definition
Classification head	It consists of one or multiple layers attached to the head of the model that outputs predictions, typically accepting embeddings and performing classification, prediction or other tasks.
Contrastive learning	A technique that can be used to improve performance of machine learning models by learning representations that bring similar samples closer to each other and move dissimilar ones further apart in the embedding space.
Decoder	Similar to the encoder, the decoder in the transformer architecture also captures the relevant context of the input data and generates an output sequence by translating the high-dimensional embedded information in a step-by-step process. It can be used for many generative tasks performed by models such as GPT, BioGPT, and CEHR-GPT.
Distant supervision	A technique in machine learning that utilizes indirect labels to generate or augment training datasets for model training.
Domain-specific transformer-based models	These specialized models have been pre-trained or fine-tuned on data from a specific domain such as biomedicine.
Embeddings	They are numerical representations of data needed to process them with machine learning algorithms. Embeddings can be generated for various kinds of objects such as words, proteins, diagnosis codes, and medications.
Encoder	As a part of the transformer architecture captures the relevant context of the input data and generates a high-dimensional embedding that can be used in many downstream tasks (such as text classification, graph node classification, and image segmentation). Transformer-based models such as BERT are based solely on the encoder.
Explainable AI (XAI)	XAI refers to the field of designing algorithms that can comprehend predictions of machine learning models, providing more insights into their decision-making process.
Generative modeling	Refers to the process of generating new samples from a certain distribution that is learned from the underlying training data. The pre-trained transformer decoder-based models can generate new text, protein sequences, structured EHRs, and images.
Graph neural networks	These are neural networks specifically designed to learn from homogeneous and heterogeneous knowledge graphs and can perform tasks such as node classification, link prediction, and graph or sub-graph classification. They learn from the underlying structure and interconnections in graphs.
Fine-tuning phase	In this phase, the pre-trained model can subsequently be tuned by an additional supervised training for a specific task using labeled data.
Large language models (LLM)	LLMs are large neural networks based on transformer architecture pre-trained on vast amounts of textual data. They are capable of understanding and generating natural language.
Machine reading comprehension task	This task aims to train algorithms to comprehend and extract relevant information from textual data. The algorithm takes in a document and a query as input. The goal is to derive the correct answer based on the provided text. One common application is span extraction, where the output consists of the relevant span of text from the document that answers the query. During query definition additional knowledge can also be utilized to improve the performance.
Machine translation	This is a task of automatically translating from one human language to another using advanced machine learning algorithms.
Pre-training phase	In this phase, the machine learning models leverage the data to learn a general representation of the underlying objects (such as text or images) in a self-supervised manner.
Self-attention mechanism	It is a technique utilized in deep neural networks to focus on all or different parts of the input while processing this input. For instance, to learn the context of each word in a sentence, the model attends to every other word. This technique has been proven as beneficial for natural language processing and other sequential data.
Self-supervised learning	It is a machine learning approach in which the intrinsic data properties are utilized to create pseudo-labels for the data itself. Subsequently, the models are trained using this self-labeled data to understand and learn the underlying patterns and relationships.
Sequence labeling task	The objective of this task is to assign labels to units of a sequence. For instance, words or tokens in a sentence can be labeled as biological concepts. Similarly, in case of protein sequences, amino acids can be assigned labels for secondary structure elements (such as alpha-helix, beta-strand, or coil).
Sequence-to-sequence learning	The models trained with this learning technique are designed to map input sequences of one domain to output sequences of another domain. Summarization or translation of text or predicting the secondary structure from protein sequence are typical examples of sequence-to-sequence learning tasks.
Relational graph attention networks	These are a type of graph neural network that, as the name suggests, apply the self-attention mechanism to graph-relational data modeling the different relationships embedded in the graph.
Representation learning	It enables the data such as text, images, protein sequences for processing with mathematical operations by representing them as compact and dense vectors. These vectors are also called embeddings that carry relevant features of input data.
Transfer learning	With this technique the model designed for one task is reused or fine-tuned to perform a different, however related task, leveraging its pre-trained knowledge to improve learning efficiency and potentially achieve better performance with less data.
Transformer	It is a deep neural network architecture that utilizes self-attention to process sequential data (such as text, protein sequences and images) in a modular and scalable encoder-decoder architecture.
Transformer-based models	Deep learning models that employ the self-attention mechanism to handle data. Their structure utilizes encoder, decoder, or both parts of the transformer architecture.
Vision transformer (ViT)	Transformer architecture specifically designed for computer vision tasks.

## Biomedical natural language processing

### Domain-specific transformers

Transformer-based models have made major strides in the biomedical NLP field, largely through adapting general language models for the biomedical domain by pre-training on huge publicly available biomedical corpora including documents from databases such as PubMed, PubMed Central (PMC), and Medical Information Mart for Intensive Care-III (MIMIC-III) [12, 13]. The majority of the studies introducing domain-specific language models often follow a familiar pattern, focusing on a specific transformer-based model architecture, initializing it with random weights or the weights of a general language model, pre-training the initialized model with domain-specific corpora as well as multiple objective tasks, and finally evaluating the models with different sizes on various biomedical downstream tasks.

For instance, BioBERT, which is initialized with the weights of the general English language model Bidirectional Encoder Representations from Transformers (BERT) [3], is a domain-specific model further pre-trained on PubMed abstracts and PMC full-text documents [14]. BioBERT was fine-tuned for various downstream biomedical NLP tasks and achieved new state-of-the-art performances for named entity recognition (NER), question answering (QA), and relation extraction (RE). More studies have introduced various variants of biomedical pre-trained models using different transformer-based architectures such as ELECTRA [15], RoBERTa [16] and GPTs (GPT1, GPT2, GPT3) [2, 9, 17]. Furthermore, BERT variants have been pre-trained on different types of biomedical corpora, see Table 2 for an overview.

**Table 2:** Overview of pre-trained biomedical language models

Study	Data sources	Model architecture	Biomedical tasks
BioBERT [3]	PubMed, PMC	BERT	Biomedical named entity recognition (NER), relation extraction (RE), and question answering (QA)
PubMedBERT [21]	PubMed	BERT	Biomedical NER, RE, QA, evidence-based medicine information extraction, document classification, and sentence similarity
BioMegatron [22]	PubMed	Megatron	Biomedical NER, RE, and QA
BioELECTRA [23]	PubMed	ELECTRA	Biomedical NER, RE, QA, evidence-based medicine information extraction, document classification, medical natural language inference, and sentence similarity
BioALBERT [24]	PubMed, PMC	ALBERT	Biomedical NER, RE, QA, evidence-based medicine information extraction, document classification, medical natural language inference, and sentence similarity
BioMed-RoBERTa [25]	PubMed, ChemProt	RoBERTa	Chemical relation classification
BioGPT [26]	PubMed	GPT-2	Biomedical RE, QA, document classification, and text generation
ClinicalBERT [27, 28]	MIMIC-III, i2b2 datasets	BERT	Identification of clinical entities, natural language inferencing
ClinicalXLNet [29]	MIMIC-III	XLNet	Identifying patient reports with prolonged mechanical ventilation and 90-day mortality
RoBERTa-MIMIC, ALBERT-MIMIC [30]	MIMIC-III, i2b2 datasets	RoBERTa, ALBERT	Identification of clinical entities
Clinical-Longformer, Clinical-BigBird [31]	MIMIC-III	Longformer, BigBird	Document classification, question answering, named entity recognition, natural language inference
GatorTron [32]	University of Florida Health, MIMIC-III, PubMed, Wikipedia	BERT, BioMegatron	Clinical concept extraction, medical relation extraction, semantic textual similarity, medical natural language inference, medical QA
Bioreddit-BERT [33]	Reddit health-related articles	BERT	Biomedical named entity recognition, adverse reaction mention detection
Bio-GottBERT [18]	German medical text	BERT	Identification of procedures, diagnoses, and medications
CamemBERT-bio [19]	French clinical documents	RoBERTa	Detection of clinical entities
KM-BERT [20]	Korean medical literature	BERT	Identification of diseases and treatment entities

Finally, different efforts have been made to develop language-specific transformer variants for biomedical texts in different regions of the world (Table 2). Some examples of these variants are Bio-GottBERT [18], CamemBERT-bio [19], KM-BERT [20] dedicated to languages like German, French, and Korean, respectively. Noteworthy, the main limitation of these efforts is often the limited availability of language-specific data.

### Applications to document and topic classification

Document and topic classification are typical NLP downstream application tasks to which pre-trained transformer models have been applied in biomedicine: During the Coronavirus disease 2019 (COVID-19) pandemic, a new search engine LitCovid [34] was introduced by the United States National Library of Medicine (NLM), which provides an overview of the latest COVID-19 literature and allows users to filter the literature based on different categories such as case reports, mechanism, prevention, or diagnosis. The classification of the literature was done manually by the creators. However, in a later stage, various experiments with transformer-based models like BioBERT, PubMedBERT, and others showed high performance with an F_1_-score of approx. 94% to automatically assign categories to new literature [35]. CO-Search is another example of a COVID-19 search engine that used a Siamese-BERT-based document retrieval engine with a strong evaluation performance [36]. Nentidis et al. [37] report results of a semantic indexing challenge in which the best participating system utilized BERT and BERTMeSH [38] models.

### Applications to Named Entity Recognition (NER) and linking (NEL)

After identifying relevant documents for a certain topic, one is often interested in finding hidden but valuable biomedical concepts inside them. NER and named entity linking (NEL) tasks are specifically designed to extract these relevant concepts and link them to biological databases. Such concepts appear in various areas of biomedicine, ranging from molecular biology (genes, proteins, microRNAs, biological functions, and cellular components) to the clinical domain (medication/drug, adverse drug reactions, diagnoses, and diseases). For instance, the sentence “Apolipoprotein E: Structural Insights and Links to Alzheimer Disease Pathogenesis” (PMID:33,176,118) contains the mention of the protein *Apolipoprotein E*, the disease *Alzheimer disease*, and the biological process *Pathogenesis* that can be linked to Uniprot term *APOE_HUMAN (ID: P02649)*, disease ontology term *Alzheimer’s disease (DOID:10,652)*, and National Cancer Institute Thesaurus (NCIT) term *Pathogenesis (NCIT: C18264)*, respectively. In the case of NER, the majority of studies have considered this task as a sequence labeling task (Table 1), in which they used BERT-based models to predict labels for each token in a sequence. Rather than a sequence labeling task, NER has also been formulated as a machine reading comprehension task (Table 1), which allows easy integration of prior knowledge into models [39].

Most authors fine-tune domain-specific transformer models, such as BioBERT, to detect one specific entity, for example drugs or genes [14]. However, multi-task learning strategies have also been proposed to detect chemical or disease mentions with one single model [40]. Some work has also been performed to capture complex cases of entities (such as discontinuous or overlapping entities) by Khandelwal et al. [41], where they combined BERT and GloVe embeddings with a new label-tagging schema to train an NER model in a distant supervision setting showing a significant performance boost in detection of disorder entities obtained from clinical free-text notes. Zaratiana et al. [42] have studied an integration of a BERT-based model with graph neural networks to create a span representation that can reduce the number of overlapping spans of disease mentions. They reported an F1 performance of 87.43%, however, the best F1-score reported on the used dataset is at 90.48%^1^. An overview of different studies employing transformers for NER and NEL is shown in Table 3.

**Table 3:** Overview of biomedical named entity recognition studies employing transformer models

Study	Data sources	Model architecture	Biomedical tasks
[43]	Emergency department notes from Stanford Health Care	BioBERT-based model	Extraction of COVID-19 symptoms and risk factors
[44]	Mental state examinations from University Hospital Aachen	GermanBERT	Extraction of psychiatric symptoms
PLM-ICD [45]	MIMIC-II, MIMIC-III	BioBERT, ClinicalBERT, PubMedBERT, RoBERTa-PM	Prediction of clinical coding of medical records
[46]	i2b2 corpora, Physionet corpus, Dernoncourt-Lee corpus	BERT, SciBERT, BioBERT	Deidentification of clinical records
[47]	EHRs, Stockholm EPR corpora,	Swedish BERT	Deidentification of clinical records
BERN2 [48]	PubMed	BioBERT	Recognition and normalization of genes/proteins, disease, drugs, species, and mutations

### Applications to relation extraction

Relation extraction, often performed after NER, is one of the main tasks in information extraction, which creates semantic links between two or more entities appearing in the text. These links, among others, can be loose (associates, interacts, correlates, etc.), quite specific (increase/decrease, binds, has participants, etc.), or even causal (directly increases, directly decreases, determined by) as defined by the relation ontology [49]. For instance, the sentence “STK38 is associated with PPARgamma” (PMID:34,670,478) contains a simple association relation between two proteins, whereas “Mitotic exit kinase Dbf2 directly phosphorylates chitin synthase Chs2” (PMID:27,086,703) describes a causal relation. The extracted relations from unstructured text are mostly used to construct biomedical knowledge graphs and expand existing ones with new knowledge [50, 51].

Transformer-based models have achieved remarkable success in extracting relations from textual content. Most of the studies have typically fine-tuned BERT-like models on subject-predicate-object relations of one dataset in a supervised manner. For instance, Zhu et al. [52] utilized BioBERT to extract drug-drug interactions from text with an overall F1 performance of 80.9% beating previous deep learning approaches. Other approaches for relation extraction involve multi-task learning, where multiple datasets are used for fine-tuning with the intuition that a model will learn a general representation of encoded relations that are of different types. To extract associations between drug-drug, chemical-protein, and medical diagnosis-treatment concepts, Moscato et al. [53] proposed a transformer-based architecture with multiple classification heads each designed to learn features for a specific type of relation. With their multi-task model, they could improve the performance by approx. 1.5% for chemical-protein and medical diagnosis-treatment associations. However, the model showed a decline of performance by 0.6% for drug-drug interactions in comparison to the single-task model. This showed that the effectiveness of multi-task learning can vary across different datasets. Solutions have also been proposed to simultaneously link entities and extract relations either by integrating multiple models in a pipeline manner [54, 55] or train a joint model responsible for extracting entities and relations at once [56–58]. Some have also experimented with datasets that were created either using distant supervision [59] or even without any supervision [60]. An overview about different studies employing transformers for relation extraction is shown in Table 4.

**Table 4:** Overview of biomedical relation extraction applications

Study	Data sources	Model architecture	Biomedical tasks
[64]	PubMed	BioPREP based on BioBERT	Detection of Xanthium compound-diabetes associations.
BioPrep [65]	SemMedDB	BioBERT, SciBERT	General biomedical predicate classification
[66]	Wikipedia and Mayo Clinic articles from DISNET	BioBERT	Creation of disease network
[67]	DrugTargetCommons, ChEMBL, DisGeNet, PubMed	SciBERT, BioBERT, BioMed-RoBERTa, BlueBERT	Prediction of drug-target interactions
KSM [68]	PubMed, BioCreative VI Track 4 PPI extraction task dataset	Multiple transformers with knowledge selector	Identification of protein-protein interactions
Patent_BERT [69]	CLEF 2020 - ChEMU Task data, Chemical Patents	BioBERT	Extraction of chemical reactions
[70]	Drug-adverse event corpus, PubMed	RoBERTa	Identification of drug-adverse effect relations
[71]	PubMed	BioBERT	Identification of plant-phenotype relations

To get an even broader view of transformer-based models used in biomedical text mining - especially on tasks this work has not focused on - we refer to various surveys published by many researchers around the world [30, 61–63].

In summary, transformer-based models are well set in the biomedical NLP field. One main challenge is however the lack of clinical datasets due to privacy reasons, which hinders the development and evaluation of models specific to clinical settings. Another challenge is the limited diversity of datasets used in studies evaluating pre-trained models as they often focus on single entity types like disease and chemical mentions. More efforts to utilize and generate NLP datasets that cover a wider range of biomedical entities and relations are required. Furthermore, the processing and analysis of longer biomedical texts still poses a challenge, which require sophisticated models. Newer models including LLaMa, BLOOM, and GPT4 implement techniques to cope with these challenges by enabling in-context learning and allowing to process longer texts. However, since these models are not specifically designed for the biomedical domain, thorough evaluation efforts are necessary to identify their advantages and limitations.

## Biological sequences

Biological sequences, such as deoxyribonucleic acid (DNA), ribonucleic acid (RNA), or protein sequences, are relatively similar to natural languages. In the same way that characters in a natural language construct meaningful words, phrases, or sentences to convey some meaning, the building blocks of sequences arranged in different combinations form structures or support specific biological functions. It is not a surprise that the recent success of transformer-based models in NLP tasks also motivated the development of dedicated models to represent and analyze biological sequences. This trend is further supported by the availability of large databases (UniProt; [72], ENSEMBL [73], GenBank [74] containing vast amounts of biological sequences that can be used to perform pre-training of transformer-based models based on amino-acid as well as DNA sequences (Table 5).

**Table 5:** Overview of biological sequencing analysis studies

Study	Data sources	Model architecture	Biomedical tasks
ProtTrans [75]	UniRef, UniParc, and Big Fantastic Database	BERT, T5, Transformer-XL, Albert, Electra, XLNet	Prediction of secondary structure and per-protein location and membrane prediction
ESM-1b Transformer [76]	UniParc	Transformer	Remote homology detection, prediction of secondary structure and tertiary contacts, prediction of mutational effects
ProteinBERT [77]	UniRef, Gene Ontology	BERT extended	Prediction of secondary structure, remote homology, fluorescence, and protein stability
MSA Transformer [96]	Multiple sequence alignments (MSA) based on UniRef	Modified transformer	Contact prediction, secondary structure prediction
ProtGPT2 [82]	UniRef	GPT2	Sequence generation, homology detection, disorder prediction,
SignalP 6.0 [97]	UniProt, PROSITE, TOPDB	ProtBERT + CRF	Detection of signal peptide types
Tranception [98]	UniProt, ProteinGym	Autoregressive transformer	Protein fitness prediction
[99]	UniProt, EVmutation	ESM-1b transformer, variational autoencoder and more	Protein fitness prediction
TMBed [100]	OPM, SignalP 6.0	ProtT5 + CNN	Prediction of transmembrane classes for each residue
ReLSO [101]	GIFFORD, GB1, GFP, TAPE	Transformer-based encoder	Designing new protein sequences
[102]	BIOSNAP, DAVIS, and BindingDB	ProtBERT + ChemBERTa	Prediction of drug-target interactions
STEP [103]	BIOSNAP [104]	Siamese ProtBERT	Prediction of protein-protein interactions

Trained protein embeddings are, among other things, used to evaluate whether the prediction of per-residue secondary structure and subcellular localization shows a similar accuracy as methods that use evolutionary information [75], the recovery of proteins along the species or gene axes is possible, the biochemical properties (such as hydrophobic or aromatic nature) of amino acids can be recovered [76], or the retrieved embeddings generalize over different protein sequence lengths [77]. For instance, Elnaggar et al. [75] used the pre-trained ProtTrans models to predict the secondary structure labels (such as alpha-helix, beta-strand, or coil) for each amino acid, reaching state-of-the-art performance on multiple datasets. Although the majority of studies that develop these pre-trained models using protein sequences employ them for various downstream classification tasks, such models can also generate de novo protein sequences with the same fundamental characteristics as the natural ones [78–82]. ProtGPT2, a protein autoregressive pre-trained language model trained on 50 million sequences, is such a model that can predict subsequent amino acid sequences given a certain context (such as a number of amino acids as input) [82]. The generated protein sequences have shown properties of globular proteins and preserved functional hotspots [82]. However, major limitations still exist as there is no way of anticipating the discovery of functional traits underlying new protein sequences, which would require costly high-throughput experimental approaches.

Recent studies have more systematically explored amino acid sequence representations learned by pre-trained transformers [83–85]. For example, the analysis by Detlefsen et al. [83] shows that pre-trained transformer models have difficulties separating details of a single protein family. In consequence, the authors propose fine-tuning (evo-tuning) on the respective protein family to increase their capacity to show clear phylogenetic separation. They also show that enforcing specific biological properties on representations is not a straightforward task and that it is currently steered by model architecture, specific preprocessing (such as using multiple sequence alignment) of underlying data, objective functions for the pre-training, and placing prior distributions on parts of the model to better mimic certain biological traits.

Fine-tuned transformer models for amino acid sequences have been used for various downstream tasks such as protein function classification, protein fitness prediction, and detection of protein interactions with chemical substances (Table 5). Furthermore, AlphaFold [86, 87] has achieved considerable improvements in the protein 3D structure prediction by using protein sequences as input. AlphaFold2 [87], builds upon two core deep learning-based modules, namely Evoformer and Structure modules, that has significantly improved the performance on the Critical Assessment of Protein Structure Prediction (CASP) 14 dataset by setting a new state-of-the-art [86, 87]. The transformer-based Evoformer module uses representation of multiple sequence alignment (MSA) and pairwise representation of protein sequence as input. The MSA, which is precomputed by conducting a search through sequence databases to find sequences that resemble the input protein sequence, informs the model about evolutionary conservation and variation. Whereas, the pairwise representation captures the interactions between pairs of amino acid residues, which is crucial for understanding the spatial geometry of protein. The Structure module uses these representations to construct an atomistic model of the protein’s structure. It employs an additional attention mechanism and optimization procedure to ensure that the predicted 3D structure is physically plausible and adheres to known biophysical constraints. AlphaFold2 combines both modules to refine the representations and 3D structure prediction in an iterative process to produce the final structure. Like AlphaFold, the transformer-based models RoseTTAFold [88] and ESMFold [89] were independently developed to also predict accurate 3D-protein structures by learning patterns appearing in protein sequences.

Some recent studies have proposed to learn and capture global representations of DNA sequences [90, 91]. Ji et al. [91] pre-trained a DNABERT model, which is based on the BERT model with masked language modeling (MLM) objective and used tokenized k-mer (with best k at 6) sequences as input instead of regarding each nucleotide as a single token. Due to the specific tokenization, the vocabulary size of DNABERT was set to 4^k^ + 5 (using permutations of 4 nucleotides with additional 5 special tokens such as for separator and padding). The pre-trained DNABERT model was analyzed using various fine-tuning tasks showing particularly that it can effectively identify proximal and core promoter regions, transcription factor binding sites, and functional genetic variants. Furthermore, DNABERT can also be used for interpretability by using learned attention weighting that characterizes the contextual relationships within a sequence to visualize its important regions and motifs.

Another relevant biological problem of how non-coding DNA regions influence gene expression in cells has been analyzed by Avsec et al. [92], who propose a transformer-based architecture called Enformer that enables the integration of long-range interactions in the genome producing significant improvements in predicting tissue and cell-type-specific gene expression. Similar to Kelley et al. [93], to read long sequences with a size of around 197.000 base pairs, the Enformer uses a number of convolutional layers that perform convolution on input sequences to reduce the spatial dimensionality. After the convolutional layers, instead of dilated convolution as used by Kelley et al. [93], the Enformer implements transformer layers that use attention mechanisms to represent the long-range interactions. The Enformer has shown significant performance gains in gene expression prediction; however, it has not yet reached the accuracies of experimental approaches. Furthermore, Enformer has also shown improvements in variant effect prediction that was performed on expression quantitative trait loci (eQTL) data [92].

In summary, studies on pre-trained transformer-based models for biological sequences have highlighted their capabilities to produce state-of-the-art results on 3D structures, functions, and interactions prediction. These sequence models however have similar limitations to NLP models. They require huge amounts of training data, which can represent a bottleneck for certain sequences (such as small RNAs). Additionally, these models often struggle to capture long-range interactions due to fixed-length context windows, which can be crucial in biological sequences. In the case of protein structure prediction, AlphaFold and others are highly accurate in predicting single protein chains; they however lack the ability to generate precise multi-chain protein complexes. Newer studies such as AlphaFold-Multimer [94] and ESMPair [95] have extended the previous models to also predict accurate protein complex structures. While transformer-based models show the ability to generalize on biological sequences data, further research is required to identify additional methods (for e.g. layers or architecture) to overcome the aforementioned limitations.

## Structured-longitudinal electronic health records

Electronic health records (EHRs) are now routinely and in vast quantities collected by many healthcare systems. Typically, they contain unstructured information like clinical notes but also structured data, including time-stamped diagnosis and medication codes as well as time-stamped codes for medical procedures. The latter provide excellent opportunities for the efficient development of machine learning models for better personalized healthcare. However, it is difficult to utilize such data due to high dimensionality, heterogeneity, temporal dependency, sparsity, and irregularity [105]. More specifically, structured EHRs can be regarded as an instance of multivariate discrete, irregular time series data.

Several studies have recently proposed transformer-based models for the analysis of structured EHR data. The intuition behind these approaches is that sequences of diagnosis, procedure and medication codes might be interpreted as a kind of language, in which codes recorded at one particular visit might be viewed as tokens. Accordingly, transformers have been pre-trained on large amounts of patient data to generate numeric representations of a patient’s medical history, which are then used for downstream tasks like medication recommendation or mortality prediction. For example, Shang et al. [106] developed the graph-augmented transformer model G-BERT. It uses the hierarchical information from the International Statistical Classification of Diseases and Related Health Problems (ICD) and Anatomical Therapeutic Chemical (ATC) ontologies to train a graph neural network, which encodes in a first step diagnosis and medication codes in a lower dimensional space. In a second step, corresponding concept embeddings are used as a modified position encoding in a BERT-like transformer architecture. The authors pre-trained their model on 20,000 patients from the MIMIC-III dataset, then applied it to a medication recommendation task and found it slightly superior to baseline techniques (1.06% gain in AUPR to the second-best approach).

Later, Li et al. [107] developed BERT for EHR (BEHRT), which uses an altered embedding layer to process a sequence of diagnosis codes. Unlike G-BERT, the model provides a patient representation for the entire medical history rather than each visit. When applied to a diagnosis code prediction task, BEHRT surpassed baseline methods (1.2–1.5% higher area under the receiver operating characteristic curve (AUROC) and 8.0-10.8% increased area under the precision-recall curve (AUPR) for the disease prediction task). Since BEHRT – like many other transformer-based models – is restricted to a maximum sequence length of 512 codes, Li et al. [108] devised a hierarchical BEHRT (HI-BEHRT) variant in a subsequent study. This method applies BEHRT to parts of the medical history using a sliding window separately before aggregating the information by forwarding the individual representations to a final transformer. In addition to the hierarchical modification, the authors included information on medications, procedures, and laboratory tests. In disease prediction tasks, it was discovered that HI-BEHRT outperforms BEHRT by 1–5% and 3–6%, respectively, in terms of AUROC and AUPR. Another variant is the Med-BERT model [109]. Compared to G-BERT and BEHRT, it employs a more extensive vocabulary of diagnosis codes. Furthermore, it introduces a new training objective called prediction of prolonged length of stay (LOS). During pre-training, the model predicts whether patients had hospital visits of seven or more days (LOS > 7 days) for their entire EHR sequences. After pre-training on data from 28 million patients, the model was applied to a disease prediction task. On three datasets originating from two clinical databases, the AUROC performance was increased by 1.21–6.14% compared to the baseline approaches. Very recent work further extended Med-BERT by adding demographic information, medications as well as quantitative lab measurements [110].

Other studies addressed the potential shortcomings of the approaches mentioned above. For instance, Pang et al. [111] proposed CEHR-BERT that, unlike Med-BERT and BEHRT, employs a different method to embed the time-series data before passing it to the transformer layers. It uses embeddings initialized with time2vec model [112] to encode the relative time between visits and the patient’s age. The age, time, and concept embeddings are concatenated and passed through a fully connected layer to generate the BERT architecture’s temporal concept embeddings. In addition, it incorporates a new pre-training task called visit type prediction (VTP) alongside MLM. This task requires the model to determine if the visit was inpatient, outpatient, emergency, or masked. Compared to baseline approaches, including the retrained versions of BEHRT and Med-BERT, CEHR-BERT increased AUPRs and AUROCs by 0.6–4.2% and 0.4–2.51%, respectively. The aspect of appropriate time encoding was also covered in several other studies [113–117].

In contrast, Agarwal et al. [118] based their Transmed approach on the notion of a hierarchical transformer for EHRs. On the one hand, a static context encoder was employed to handle information such as a patient’s age, sex, race, and prior conditions such as diabetes or smoking. On the other hand, temporal context encoders were used to process the information at individual visits. The aggregated representations from the static and temporal encoders are then used to predict a patient’s risk of hospital stay or mechanical ventilation following a COVID-19 diagnosis. Across all four tasks, Transmed outperformed a newly pre-trained version of BEHRT (11–20% higher AUROC) and was mostly on par or better than a baseline gated recurrent unit (GRU) model.

There have also been attempts to combine structured and unstructured EHR data into joint patient representations. For instance, the Bidirectional Representation Learning model with Transformer architecture on Multimodal EHR (BRLTM) [119] utilizes diagnosis, drug, and procedure codes as well as information derived from unstructured clinical notes via latent Dirichlet allocation (LDA). When the authors compared BRLTM to other models, including a retrained version of BEHRT, they discovered that it was superior at accurately predicting diseases over multiple time frames. Liu et al. [120] followed a different approach with their Med-PLM model. Instead of deriving features from clinical notes, they use ClinicalBERT for processing clinical notes and a G-BERT-like model for processing structured EHR data before combining both using a cross-attentional module. The authors found that the final model outperformed unimodal counterparts (e.g., ClinicalBERT or G-BERT) in all tasks, highlighting the potential of merging both data modalities. Similarly, Darabi et al. [113] used both data modalities for their TAPER model and reached comparable results.

Another recent development in the context of EHRs is the synthetic generation of EHRs with transformers. Cheng et al. [121] recently proposed CEHR-GPT, a model that builds upon their previous work on CEHR-BERT to generate synthetic EHRs using GPT. Unlike CEHR-BERT, CEHR-GPT includes additional information on demographics, patient history, and temporal dependencies. Each visit is represented by a visit type token (VTT), and time is encoded using artificial time tokens (ATT) and long-term (LT) tokens. In their experiments, the authors compared three different patient representations of GPT. They found that CEHR-GPT was the most suitable variant for generating realistic synthetic EHR data while preserving patient privacy and temporal dependencies. However, they reported that the prevalence of concepts in the generated data was skewed compared to the original data and that the representation of time intervals is currently limited, suggesting that further improvements could be made in training the model and the representation of EHR data.

A broad overview of different studies employing transformers for structured-longitudinal EHR analysis is shown in Table 6.

**Table 6:** Summary of models performing structured-longitudinal EHR analysis

Study	Data sources	Model architecture	Biomedical tasks
BEHRT [107]	CPRD	Transformer-based encoder	Disease prediction
Hi-BEHRT [108]	CRPD	Hierarchical BEHRT	Disease prediction
G-BERT [106]	MIMIC-III	Graph neural network and BERT	Drug recommendation
BRLTM [119]	UCLA EHR data	Transformer-based encoder	Disease prediction
Med-BERT [109]	Cerner Health Facts®, Truven Health MarketScan®	Transformer-based encoder	Disease prediction
ExMed-BERT [110]	IBM Explorys Therapeutic Dataset	Extended Med-BERT	Disease prediction
CEHR-BERT [111]	CUIMC-NYP OMOP	Transformer-based encoder with additional FFN for temporal embedding	Various predictive tasks (disease, readmission, death, hospitalization)
Med-PLM [120]	MIMIC-III	G-BERT / Med-BERT + ClinicalBERT + Cross-modal module	ICD coding, readmission, drug recommendation
TransMED [118]	STARR OMOP	Hierarchical use of BERT	Hospital stay, ventilation risk
T^3^Net [114]	KPMAS	Transformer-based encoder	Hospitalization and mortality prediction
TAPER [113]	MIMIC-III	Transformer-based encoder, BERT	Readmission and mortality prediction
CEHR-GPT [121]	EHR data from the Columbia University Irving Medical Center-New York Presbyterian Hospital	Transformer-based decoder, GPT	Generation of synthetic EHR data

In summary, transformer-based models are promising for working with structured EHR data. However, applying these models to EHR data also presents several challenges. Firstly, EHR data is highly heterogeneous and diverse, making it relatively unclear how to best represent it compared to text, sequence, and image data. Many studies focus on finding a suitable data representation. In addition to this challenge, comparing these models and their results is difficult. Since most pre-trained models and datasets are publicly unavailable due to privacy concerns, a direct comparison of the models is often impossible. Although studies often use other models as baselines and perform pre-training on available data to compare model architectures, a direct comparison of initially pre-trained models is not feasible, as is common in the NLP field. Furthermore, Kumar et al. [122] point out that simple linear models could not only be data and computationally efficient but could achieve comparable performance to transformer-based models. For instance, they propose an attention-free architecture called SANSformer that outperformed BEHRT and BRLTM models. Despite these challenges, transformer-based models remain a promising tool for analyzing EHR data. Further research is important to understand their full potential as well as limitations and how they can improve patient outcomes and provide better decision support.

## Biomedical images

Due to the self-attention mechanism employed in transformer-based models, they have shown superior ability to model long-term dependencies in data, however, mostly in cases where the data is of sequential nature. Recently, transformers have also been adapted successfully to a wide variety of image analysis cases. For the purpose of image analysis, the image is first split into a sequence of patches (regions), which are then flattened to fixed vector length - quite similar to tokens. The flattened image patches are then linearly projected and combined with their positional embeddings that provide spatial information on each patch. The sequence of transformed patches can then be fed to a transformer. This approach is referred to as a vision transformer (ViT) in the literature [123–126]. Dosovitskiy et al. [124] formulated image classification as a sequence prediction task, which he addressed via a ViT. They examined two approaches for aggregating spatial information from images: the use of a CLS token and global pooling [124]. The CLS token in ViTs aggregates global information through self-attention, dynamically adjusting to capture complex image relationships. Global pooling, including methods like global average and max pooling, simplifies feature aggregation by applying straightforward mathematical operations across all image patches. While the CLS token’s aggregation is learnable and adapts to task specifics, global pooling offers a more generalized and computationally efficient summary [124].

ViTs have been applied to medical images derived from imaging techniques such as X-ray, computer tomography (CT), MRI, ultrasonography, optical coherence tomography (OCT), and high-content cell imaging screens. For instance, ViTs were used to analyze lung X-rays to detect COVID-19 disease [127–129], breast sonography images to classify breast cancer [130, 131], or femur X-rays to check for fractures [132]. Chen et al. [133] have proposed a ViT to detect gastric cancer from histopathological imaging data. Furthermore, CT images were used by Wu et al. [134] to build a medical application for classification of emphysema that can be further divided into three different subtypes, whereas Wang et al. [135] screened rare medical OCT imaging dataset for lesions associated with genitourinary syndrome of menopause. Nonetheless, MRI datasets have also been classified using ViTs for brain tumors [136] or for intraductal papillary mucosal neoplasms in the pancreas by [137]. Upon closer examination of the work of Tanzi et al. [132], you can observe potential benefits of ViT architectures compared to conventional approaches. Based on their results, it seems worth exploring the superiority of embedding space representations generated by ViTs, which can boost performance for medical classification tasks. Examining attention layers, that are commonly part of ViT architectures, makes these models inherently explainable, an attribute highly regarded by clinicians for model evaluation. Lastly, their retrospective analysis of integration into clinical practice, allows for the conclusion that a ViT-based computer aided diagnosis (CAD) system can contribute to improving clinical workflows and decision making for young residents and experienced doctors alike.

Another relevant task in the biomedical computer vision field is to detect segments of object instances such as lesions in functional magnetic resonance images, tumors in histopathological images, brain tissues in magnetic resonance images, retinal vessels in fundus imagery, or single-cell information from microscopy imagery [138, 139]. Transformer-based models are being heavily used for segmentation as they often improve accuracy compared to the traditional convolutional neural network-based (CNN) methods. Although most studies use hybrid transformer architectures, some have also built pure transformer-based models. For instance, Gao et al. [140] have proposed a hybrid transformer-based architecture UTNet by integrating a complexity-reduced self-attention into a CNN for segmentation. In comparison Huang et al. [141] introduced the pure transformer-based method MISSFormer, optimized especially for medical image segmentation tasks. Most studies have focused on the medical field, but some have also applied transformer-based methods for segmenting cells in images that originated in in-vitro experiments. Prangemeier et al. [139] have proposed a cell detection transformer for direct end-to-end instance segmentation, reaching a similar accuracy as the CNN-based methods while showing the simplicity and improved runtime of the proposed model.

In the drug discovery field, it is common nowadays to perform an automated high-content screening of cells treated with specific chemical substances. These screening experiments might identify substances that have desirable effects on the phenotypes of cells. High-content images of cells are also used for image-based profiling, where the profiles are derived by extracting relevant features from screened images [142]. Such phenotypic profiles can be used in downstream applications such as identifying a disease-associated phenotype, identifying lead compounds, bioactivity and toxicity assessment, and detecting a compound’s mechanism of actions [142], where recently transformer-based models are being applied to [143, 144]. For instance, Cross-Zamirski et al. [143] proposed a ViT-based model that uses weak labels to learn phenotypic representations from a publicly available dataset containing high-content images of cells and evaluate the model on two mechanism-of-action classification tasks. Furthermore, the authors show that the representations are biologically meaningful by analyzing the attention maps. Table 7 provides a broader overview of recent applications of ViTs.

**Table 7:** Summary of transformer models for biomedical image analysis

Study	Data sources	Model architecture	Biomedical tasks
[127–129]	Lung X-rays	Vision transformer	Detection of COVID-19
[130, 131]	Breast sonography images	Vision transformer	Classification of breast cancer
[132]	Femur X-rays	Vision transformer	Detection of fractures
GasHis-Transformer [133]	Histopathological imaging	Vision transformer	Detection of gastric cancer
[134]	CT imaging	Vision transformer	Classification of emphysema and their subtypes
ViT-P [135]	OCT imaging	Vision transformer	Detection of lesions associated with genitourinary syndrome of menopause
[136]	MRI dataset	Vision transformer	Detection of brain tumors
[137]	MRI dataset	Vision transformer	Classification of intraductal papillary mucosal neoplasms in the pancreas
Cell-DETR [139]	Live-cell microscopy dataset	Based on transformer encoder-decoder	Segmentation of yeast cells in microstructures
[143]	Stained breast cancer cell images	Vision Transformer	Mechanism of action prediction of cells treated with compounds
[144]	High-content imaging screen	Vision Transformer	Classification of cell phenotypes

Even though ViTs have proven to be powerful architectures for a variety of problems in biomedical imaging, they can not be recommended unlimitedly in favor of more established computer vision models, e.g. convolutional neural networks (CNNs) [145, 146]. It is important to understand how both architectures “perceive” images, in order to understand its particularities, advantages and disadvantages. The architecture of convolutional networks is inspired by the visual cortex of the brain [147]. They use receptive fields to learn kernels enabling them to recognize features crucial to their task. A subsequent pooling operation relatively increases the receptive fields of the kernels. This process is repeated iteratively, so the kernels can interpret more distant areas of the image [148]. This, by design, creates inductive biases, like translation equivariance and locality [124], important properties for image classification.

In contrast, as described earlier, a ViT treats an image as a sequence of patches, and through self-attention, every patch of the sequence attends to every other patch, so it needs to learn all spatial relations from data training [124]. Essentially, this causes ViT to struggle in effectively generalizing with limited data [124]. However, their performance scales well with growing datasets, outperforming CNNs as the number of training samples increases. Unfortunately, especially in the biomedical domain, large publicly available datasets are scarce.

Nonetheless recent work by He et al. [149] has shown that pre-training techniques, such as training a masked autoencoder (MAE) for patch embeddings, can reduce the number of training samples, training times, and boost performance in natural images. Zhou et al. [150] later showed that this can be applied in the medical domain as well. Varma et al. [151] tackled the issue of ViTs relying on predefined image sizes, necessitating pre-processing steps that can degrade image information. Through their flexible positional embedding and alternate batching strategies, they can reduce image manipulation while maintaining fine-grained image features.

Driven by its popularity and constant developments through ongoing research, one can assume that ViT architectures will increase in value and impact for biomedical imaging tasks in the near future.

## Biomedical graphs

Besides textual content, biological sequences, imaging data, and structured EHR data, graphs are frequently used in biomedicine to describe relations between concepts. Graphs can cover various aspects of life sciences, hence, they can connect different types of nodes and edges with each other. Graph representation learning with machine learning methods enables the usage of graphs for various biomedically relevant downstream tasks such as protein-protein interaction prediction, prediction of adverse drug reactions, cell-type-association prediction, disease-subgraph classification drug-interaction prediction, patient-treatment prediction [152]. These tasks can be modeled as graph or sub-graph classification, node classification, or link prediction, which are often performed by encoding the information included in graphs, such as the graph structure, local graph neighborhoods, and the distinguishing features of nodes and edges [152].

Graph Transformer [153], Graph Transformer Networks [154], GTransformer [155], Structured Transformer [156], GraphFormers [157], and Relphormer [158] are some adaptations of transformer-based models suitable for graph representation learning. Transformers for graphs are conceptually similar to relational graph attention networks (RGATs) [159]. They regard each node of a graph as an entity in a pseudo-sequence. However, unlike transformers for sequences, the attention is restricted to neighboring nodes, hence taking into account the graph topology.

Graph-based transformers have, for instance, been applied in the drug discovery field where the focus lies on the identification of targets [160, 161], prediction of response [162], prediction of ATC code [163], or adverse reactions for a certain drug [164]. Additional work has also been performed to predict the properties of molecules involving toxicity, carcinogenicity, or blood-brain barrier penetration [165–167]. Recently, also textual and image analysis tasks (such as relation extraction or deformable image registration) have been successfully pursued using graph transformers [168, 169]. A further example is the prediction of interactions between transcription factors and DNA, which can be formulated as a link prediction task in a bipartite graph [170]. Other authors have delved into engineering new proteins by generative graph representations of 3D protein structures [156, 171]. Also, the prediction of protein-protein interactions has been performed using graph neural networks while using protein 3D structure graphs and learned sequence embeddings of ProtBERT [172].

Although the analysis or usage of graphs to support biomedical tasks with graph transformers is yet focused only on some niche areas, researchers are already prospecting new fields, such as the analysis of single-cell multi-omics in immuno-oncology to characterize cellular heterogeneity [173], where this technology could also be helpful. Nonetheless, further experiments are required to assess whether the transfer learning strategies, along with graph transformers, will ultimately prevail over the general graph neural networks like RGATs.

## Transformers for multimodal data

The majority of existing research studies have addressed biomedical tasks using just one single data modality, however, modeling complex processes of biology and medicine inherently requires the integration of and learning on multiple modalities, such as genetic, proteomics, pharmacogenomic, imaging, and text [174]. Recently, transformer-based models have been adapted to process multiple data modalities simultaneously. Koorathota et al. [175] introduced a multimodal neurophysiological transformer for recognizing emotions using multiple modalities (such as time series and extracted features) obtained through electroencephalography, galvanic skin response, and photoplethysmogram techniques. Inspired by the multisensory integration mechanism of the brain, Shi et al. [176] proposed an adapted transformer-based model to integrate visual and auditory modalities to improve emotion and bird species recognition using video-audio clips.

Furthermore, vision-and-language models are a recent development, which take textual content and images as input and jointly learn to capture the relationships between both modalities. These models have also been adapted to the clinical domain, for instance, for chest X-ray disease diagnosis [177] or to automatically generate reports for abnormal COVID-19 chest CT scans [178]. Similarly, paired images and textual reports of chest and musculoskeletal X-rays were used with contrastive learning to build new pre-trained models that improved upon medical image classification and retrieval on various datasets [179]. Others have explored an integration of molecular structures using simplified molecular-input line-entry system (SMILES) signatures in biomedical text to build a transformer-based multimodal system that can predict molecular properties, classify chemical reactions, and improve NER as well as relation extraction [180]. Finally, Lentzen et al. [110] proposed a multimodal transformer architecture to combine structured EHRs with quantitative clinical measures. Their idea was a concatenation of the latent representation learned by the transformer encoder with a feature vector representing quantitative data. The concatenated representations are then passed forward through the classification head during the fine-tuning phase.

Development of transformer-based models capable of learning from multimodal data is a non-trivial challenge. These models are highly specific to the particular modalities (for e.g. text, image, or structured EHR) and tasks at hand. There is a pressing need for further investigation into how transformer-based architectures can evolve into universal architectures that are agnostic to various biomedical modalities and the underlying tasks.

## Making transformers explainable

Specifically in biomedicine, it is essential to study which features a model used to make predictions in order to identify potential flaws and build trust in the results. Since the first appearance of transformer-based models, several studies have proposed different approaches for post-hoc model explanation based on techniques developed in the booming field of Explainable AI (XAI) [181].

Most approaches focus on the implicitly learned attention weights of transformer-based models. For instance, Vig [182] developed the BertViz tool for displaying the attention weights for analysis and debugging purposes. Later, Ji et al. [91] used similar visualizations for their pre-trained DNABERT model. Following the evaluation, the authors studied the attention landscape and found, for instance, that the model prioritizes intronic sequence sections when predicting splice sites. Similarly, Avsec et al. [92] investigated their model, which was built to predict gene expression and chromatin states using the average attention weights. Their analysis revealed that the developed model attends to parts of the sequence located up to 100 kb from the gene site. A slightly different strategy was followed by Koorathota et al. [175], who proposed a multimodal neurophysiological transformer for predicting valence and arousal as a response to music. They created a metric known as the sum of absolute activation differences to interpret the interactions between the different modalities. Unlike the majority of attention-weight analyses, this analysis is neither affected by individual samples nor the selection of attention layers or heads. The study revealed that, for instance, electroencephalography and photoplethysmogram signals significantly affect the model’s prediction.

Other studies investigate the application of general-purpose XAI methodologies. For instance, Kokalj et al. [183] introduced TransShap, an adaptation of Shapley Additive Explanations (SHAP) [184] that may be utilized to evaluate and understand the functioning of text classifiers. Lastly, Madan et al. [103] applied the integrated gradients method [185] instead of focusing on the attention weights of the model. The authors utilized this method to explain their predictions on virus-host protein-protein interactions while discovering sections of sequences that contribute to the model’s predictions. Advances in the field of XAI methods, in general, have opened up new opportunities to interpret the models while gaining new insights on predictions, although significant limitations still exist due to the lack of validation datasets, hence, careful investigation of the reliability of these XAI strategies is highly necessary [186]. Furthermore, a general caveat is the possible misinterpretation of XAI approaches as providing a causal understanding of the prediction problem.

## Discussion

### Strengths of transformers

Transformer-based models have pushed the boundaries for processing and analyzing various data modalities such as text, EHRs, biological sequences, images, and graphs across a wide variety of biomedical tasks, as demonstrated by the examples shown in the previous sections. Since transformers originated in the NLP field, the biomedical NLP has seen a certain momentum with these models earlier than other disciplines, resulting in a greater number of transformer-related research studies within the NLP field. At the moment, transformers have mainly been applied to discrete data, but also first adaptations to continuous time series data have been proposed [187].

The success of the transformer can be mainly explained by two factors:

- the attention mechanism, which allows for capturing long-range dependencies in the input.
- the self-supervised learning paradigm that supports pre-training from huge amounts of unlabeled data and subsequent fine-tuning / transfer-learning of a domain-specific task.

Specifically, the second aspect allows for effective utilization of background information, which explains the often-observed superior prediction performance compared to more conventional machine learning approaches.

### Challenges when using transformers

The pre-training of transformers using the self-supervised learning paradigm depends on huge training datasets. Accordingly, the training of transformers is computationally intensive. It should be noted that transformers have millions of parameters (one of the largest model PaLM published by Chowdhery et al. [188] has 540 billion parameters), and the underlying attention mechanism has a quadratic time complexity with regard to the input sequence length. To overcome these challenges, new solutions have been proposed such as optimizing the transformer model [189–193] or applying knowledge distillation technique [194]. For instance, Kitaev et al. [191] proposed the Reformer model that improves the efficiency of the transformer by reducing the complexity of the dot-product attention mechanism and by optimizing the storage of activations in the model.

### Future direction 1: knowledge integration

Another line of research focuses on the utilization of background knowledge during the training procedure. For example, in the NLP field K-BERT is an extension of BERT, in which the input token stream is expanded by background information extracted from a knowledge graph [195]. ERNIE uses two encoders, a T-encoder for the original tokens and a K-encoder for entities in a knowledge graph, and both representations are fused [196, 197]. While the authors of these papers report enhanced prediction performances of NLP-related tasks outside the biomedical domain, there is the question of how according methods might be impacted by incompleteness and errors in the knowledge graph, which could be a major concern in the biomedical field. Furthermore, not all knowledge can be effectively represented as a graph. Depending on the respective application, other knowledge representations, such as logical rules and mathematical equations, could be worthwhile to consider in future research as well.

### Future direction 2: multimodal data integration

Integrating multimodal data is key for many systems and precision medicine tasks. Heterogeneous information across different data modalities, such as genetics, epigenetics, proteomics, metabolomics, imaging, text, and clinical observations, must be aligned and fused to perform multimodal learning with transformer-based models. Although first publications are now focusing on multimodal transformers (see section above), this line of research is still at the beginning. For example, one general challenge in the area of multimodal data integration are varying dimensions and numerical ranges of input modalities [198]. Recent studies have begun to explore general-purpose architectures that can handle different modalities of varying dimensionalities [199–201], but we expect more work to come along those lines.

### Future direction 3: generative modeling

More recently, generative transformer models have shown impressive advancements in the NLP field. One of the most prominent examples, which is however not particularly devoted to biomedicine, is ChatGPT [202]. ChatGPT has shown remarkable performances on generating near-human level textual content and leading dialogues with humans. Generative transformer models such as ChatGPT or its freely available variants (e.g., GPT4All; [203]) could in the future support many tasks in medical routine, such as generating synthetic clinical notes [32], writing discharge letters, or coding and billing diagnosis and medications. Furthermore, these models could also support the field of biomedical research. Researchers have already started experimenting with generative transformers to generate synthetic protein sequences [82, 204]. However, a huge challenge of applying such models in biomedicine is to verify the trustworthiness of the generated content. For instance, engineered protein sequences need to be experimentally tested. Automatically generated discharge letters have to be validated manually.

### Future direction 4: better explainable models

By being able to explain and understand the predictions through XAI techniques, trust and confidence can be built in biomedical AI models, which is even more relevant for decision-making processes in the clinical domain. Several general-purpose XAI techniques have been adapted for transformers recently [205–207]. Some have shown that trust in models can also be increased by producing counterfactual explanations that show under which hypothetical changes to the input a different output will be generated, a method often used by humans to understand unfamiliar processes [208, 209]. However, the XAI field as such is still in its infancy. For example, there is no generally accepted definition of “explainability”, and there is a lack of gold standards against which new methods could be compared. Accordingly, existing attempts to make transformers explainable have to be seen relative to the advances of the XAI field as a whole. While first approaches in the XAI field mainly focused on images, the development of general-purpose model explanation techniques, such as SHAP is still relatively recent. We can thus expect that with the increasing advances of the XAI field also better explanation techniques for transformers will become available.

## Conclusion

Transformers, originally created in the NLP field, are still a relatively new deep learning approach. Recent years have witnessed a dramatically increased use for various data types with transformers, which are of relevance in biomedicine, including structured EHRs, graphs, images, and biological sequences. The main strengths of transformers are the in-built attention mechanism and the possibility for self-supervised pre-training, which, however, requires huge datasets. Accordingly, transformers have currently found little use in domains where such datasets are not available, e.g., signals coming from wearable devices, or clinical studies and registries. Also, despite research on modeling time-series data with transformers [187], dedicated studies in biomedicine for this type of data are yet to emerge. Currently emerging directions of research include better strategies for knowledge integration, multimodal data fusion, and the adaptation of novel XAI techniques. We expect that efforts to integrate data across the entire healthcare system, such as those in United Kingdom (UK) like Health Data Research UK (https://www.hdruk.ac.uk/*)*, UK Biobank (https://www.ukbiobank.ac.uk/*)* and Genomics England (https://www.genomicsengland.co.uk/*)*, will enable an even more wide-spread use of transformers in the future.

## Data Availability

Data sharing is not applicable to this article as no datasets were generated or analyzed during the current study.

## Abbreviations

AI: Artificial Intelligence
BERT: Bidirectional Encoder Representations from Transformers
CAD: Computer aided diagnosis
COVID-19: Coronavirus Disease 2019
CV: Computer Vision
CT: Computer Tomography
EHR: Electronic Health Records
GPT: Generative pre-trained transformers
MRI: Magnetic Resonance Imaging
NER: Named Entity Recognition
NEL: Named Entity Linking
NLP: Natural Language Processing
NLM: United States National Library of Medicine
PMC: PubMed Central
ViT: Vision Transformers
ICD: International Statistical Classification of Diseases and Related Health Problems
ATC: Anatomical Therapeutic Chemical
VTP: Visit Type Prediction
GRU: Gated Recurrent Unit
LDA: Latent Dirichlet Allocation
SHAP: Shapley Additive Explanations
XAI: Explainable Artificial Intelligence

## Footnotes

1 https://paperswithcode.com/sota/named-entity-recognition-ner-on-ncbi-disease.
